# Association Between Homeschooling and Adolescent Sleep Duration and Health During COVID-19 Pandemic High School Closures

**DOI:** 10.1001/jamanetworkopen.2021.42100

**Published:** 2022-01-05

**Authors:** Joëlle N. Albrecht, Helene Werner, Noa Rieger, Natacha Widmer, Daniel Janisch, Reto Huber, Oskar G. Jenni

**Affiliations:** 1 Child Development Center, University Children’s Hospital Zurich, University of Zurich, Zurich, Switzerland; 2 Children’s Research Center, University Children’s Hospital Zurich, University of Zurich, Zurich, Switzerland; 3 Department of Psychosomatics and Psychiatry, University Children’s Hospital Zurich, University of Zurich, Zurich, Switzerland; 4 Department of Child and Adolescent Psychiatry and Psychotherapy, University Hospital of Psychiatry, University of Zurich, Zurich, Switzerland

## Abstract

**Importance:**

Although negative associations of COVID-19 pandemic high school closures with adolescents’ health have been demonstrated repeatedly, some research has reported a beneficial association of these closures with adolescents’ sleep. The present study was, to our knowledge, the first to combine both perspectives.

**Objective:**

To investigate associations between adolescents’ sleep and health-related characteristics during COVID-19 pandemic school closures in Switzerland.

**Design, Setting, and Participants:**

This survey study used cross-sectional online surveys circulated among the students of 21 public high schools in Zurich, Switzerland. The control sample completed the survey under regular, prepandemic conditions (May to July 2017) and the lockdown sample during school closures (May to June 2020). Survey respondents were included in the study if they provided their sex, age, and school.

**Exposures:**

High school closures during the first COVID-19 pandemic wave in Switzerland (March 13 to June 6, 2020).

**Main Outcomes and Measures:**

Sleep-wake patterns, health-related quality of life (HRQoL, assessed by the KIDSCREEN-10 questionnaire), substance use (caffeine, alcohol, and nicotine), and depressive symptoms (lockdown sample only; assessed using the withdrawn/depressed scale from the Youth Self Report). Multilevel regression models were used to assess sample differences and associations of health-related characteristics with sleep duration and depressive symptoms.

**Results:**

The total sample consisted of 8972 students, including 5308 (59.2%) in the control sample (3454 [65.1%] female) and 3664 (40.8%) in the lockdown sample (2429 [66.3%] female); the median age in both samples was 16 years (IQR, 15-17 years). During school closures, the sleep period on scheduled days was 75 minutes longer (semipartial *R*^2^ statistic [*R*^2^_β*_], 0.238; 95% CI, 0.222-0.254; *P* < .001) and the students had better HRQoL (*R*^2^_β*_, 0.007; 95% CI, 0.004-0.012; *P* < .001) and less consumption of caffeine (*R*^2^_β*_, 0.010; 95% CI, 0.006-0.015; *P* < .001) and alcohol (*R*^2^_β*_, 0.014; 95% CI, 0.008-0.022; *P* < .001). Longer sleep duration was associated with better HRQoL (*R*^2^_β*_, 0.027; 95% CI, 0.020-0.034; *P* < .001) and less caffeine consumption (*R*^2^_β*_, 0.013; 95% CI, 0.009-0.019; *P* < .001). In the lockdown sample, an inverse association was found between depressive symptoms and HRQoL (*R*^2^_β*_, 0.285; 95% CI, 0.260-.0311; *P* < .001) and a positive association was found with caffeine consumption (*R*^2^_β*_, 0.003; 95% CI, 0.000-0.008; *P* = .01).

**Conclusions and Relevance:**

In this survey study, 2 opposing associations between school closures and adolescents’ health were identified: a negative association with psychological distress and a beneficial association with increased sleep duration. These findings should be considered when evaluating and implementing school closures. Furthermore, the findings provide support for delaying school start times for adolescents.

## Key Points

**Question:**

Were sleep gains among adolescents during COVID-19 pandemic high school closures associated with better health-related characteristics?

**Findings:**

In this survey study of 8972 adolescents from Swiss high schools, during the COVID-19 lockdown, participants slept significantly longer and had better health-related quality of life and less caffeine and alcohol use than before the pandemic. Longer sleep duration was significantly associated with better health-related characteristics, although this was offset by an association of depressive symptoms with worse health-related characteristics and increased caffeine consumption.

**Meaning:**

In this study, sleep gains were associated with better health-related characteristics among youths, but depressive symptoms were associated with a worsening of the same health-related characteristics.

## Teaser

This survey study investigates the associations between sleep behaviors and adolescents’ health-related characteristics during COVID-19 pandemic high school closures in Switzerland.

## Introduction

In spring 2020, the worldwide COVID-19 pandemic was declared. To limit the spread of infection, social contacts were markedly reduced. Many countries were forced to shut down most of public life, including schools. Thus, children and adolescents experienced extreme changes in daily life. Critical concerns about the negative effects of closures were raised and have since been supported by empirical evidence. The prevalence of mental health problems in adolescents increased substantially during lockdown.^1,2^ For example, increases in depressive symptoms and anxiety and decreases in life satisfaction and health-related quality of life (HRQoL) were observed in longitudinal studies.^3,4^ Furthermore, adolescents were less physically active and spent more time being sedentary in front of screens.^5,6,7,8,9^ Findings regarding substance use have been mixed; in general, fewer adolescents consumed substances such as alcohol during the lockdown, but the frequency of consumption seemed to increase.^10^

Although, to our knowledge, there has been no controversy about the negative association of lockdown with adolescents’ well-being,^11^ a potentially conflicting finding has also appeared consistently; adolescents slept significantly longer during school closures than before them.^5,6,7,8,12,13,14^ Sleep plays a crucial role in mental and physical health,^15,16,17^ and thus, a positive association of increased sleep with adolescent well-being and health could be expected. Studies investigating later school start times (SSTs) in the morning have shown this association; early SSTs conflict with adolescents’ late biologic sleep phase and therefore contribute to the well-known chronic sleep deficit of many adolescents by preventing later wake times that compensate for biologically related delayed sleep times.^15,16,18,19^ Studies investigating the consequences of later SSTs have consistently found longer sleep duration together with positive associations with adolescents’ health.^16,19,20,21^ The sleep gains observed during school closures can be interpreted similarly: because students in homeschooling do not need to commute to school, school closures create a naturalistic delay in SSTs. Thus, adolescents appear to have benefitted from school closures that aligned their sleep schedule better with their biologic sleep phase, allowing more sleep.^5,6,7,8,12,13,14^ However, to our knowledge, whether the longer sleep duration during school closures was associated with health benefits, as in SST delay studies,^16,19,20,21^ has not been investigated.

In this survey study, we aimed to fill this research gap and investigate the association of sleep with adolescents’ health during school closure. By comparing a sample of adolescents examined during the lockdown in Switzerland in spring 2020 with a control sample examined under regular conditions,^22^ we aimed to further characterize adolescents’ sleep behavior and the health characteristics of HRQoL and substance use during school closures. We also investigated the differential associations of sleep and depressive symptoms with adolescents’ health. We aimed to render a more comprehensive picture about the outcomes of pandemic high school closures by considering the closures’ associations with both psychological distress and later SSTs.

## Methods

### Participants

In this survey study, 2 samples of respondents from nearly identical online surveys were compared. As confirmed by the ethics committee of the canton of Zurich, the study did not fall within the scope of the Swiss Human Research Act because both surveys were anonymous; therefore, ethical approval and informed consent were not required. In both surveys, all public high schools in the canton of Zurich, Switzerland, were asked to electronically circulate a link to the survey (completion time approximately 10 minutes) among their students. The control sample (details published elsewhere)^22^ included students who completed surveys from May to July 2017, and the lockdown sample included students who completed surveys from May to June 2020 during high school closure owing to the first COVID-19 wave in Switzerland. Of importance, because season affects sleep,^23^ both surveys were administered in the spring. Exclusion criteria were no information about sex, age, or school; attending a private high school; and currently experiencing COVID-19 symptoms. School start time was between 7:30 and 8:05 am in the control sample but was unknown for the lockdown sample because of substantial heterogeneity of homeschooling implementation across schools. This study followed the American Association for Public Opinion Research (AAPOR) reporting guideline; however, because it was unknown how many students received the survey invitation from their school, it was not possible to report disposition codes and outcome rates as suggested by the AAPOR reporting guideline. Instead, participation rates (ie, the percentage of participants among all high school students) were calculated; median participation rates were estimated as 38% (IQR, 35%-43%) in 2017 and 23% (IQR, 5%-42%) in 2020. Participants were compensated with participation in a raffle.

### School Closure in Switzerland

High schools in Switzerland were closed from March 13 to June 6, 2020, although starting from May 11, 2020, in-person lessons with a maximum of 5 people were allowed. The government strongly recommended staying at home and minimizing social contact, but a curfew was never in place.^24^

### Measures

The surveys were created in LimeSurvey.^25^ All questions were presented in German, and participation was voluntary and anonymous. Details about questions and answer formats are provided in eTable 1 in the Supplement.

### Sample Characteristics

Students indicated their sex, age, and primary language (Swiss German vs other). In addition, they reported whether they had a chronic physical or mental disease (yes or no) and which school they attended.

### Sleep Characteristics

The Munich Chronotype Questionnaire^26^ was used to assess sleep-wake patterns separately for scheduled days such as school days, free days such as weekend days, and alarm clock use (yes or no) on scheduled days. The bedtimes and wake times indicated by participants were used to quantify the sleep period. As in Werner et al,^22^ the sleep deficit was calculated as the sleep period on free days minus the sleep period on scheduled days.

To assess sleep difficulties, 2 questions from the School Sleep Habits Survey^27^ were used. The students indicated how many nights they had had difficulty falling asleep and sleeping through the night in the previous 2 weeks.

### Health-Related and Behavioral Characteristics

The KIDSCREEN-10 questionnaire^28^ was chosen to assess HRQoL. Depressive symptoms were assessed only in the lockdown sample with the withdrawn/depressed scale from the Youth Self Report for ages of 11 to 18 years.^29^ Raw sum scores were transformed into T values, with greater KIDSCREEN-10 values indicating better HRQoL and greater Youth Self Report values indicating more depressive symptoms. Youth Self Report withdrawn/depressed scores with T values of 70 or greater indicate clinically relevant symptom levels.^29^

Furthermore, the adolescents indicated their weekly caffeine consumption. Students older than 16 years were also asked about their weekly alcohol consumption and whether they smoked, and if so, how many cigarettes per day. In addition, students indicated how many hours per day they used digital media.

### COVID-19–Related Measures

The lockdown sample indicated whether they were in temporary homeschooling owing to pandemic school closures (yes or no) and rated on a 5-point Likert scale (1, “not at all”; 5, “very strongly”) regarding changes since the pandemic in the following areas of life: social contacts, use of digital media, and sports. Also, the participants indicated on a similar Likert scale how much they worried that the pandemic would affect their future academic achievement. To assess self-exposure and proxy exposure to COVID-19, the participants were asked whether they, someone in the same household, or someone close such as a grandparent had COVID-19 symptoms.

### Statistical Analysis

Data were analyzed using R, version 4.0.2 (R Project for Statistical Computing). Two-tailed tests were used for all analyses, and *P* < .05 was considered statistically significant. All variables had nonnormal distributions (Kolmogorov-Smirnov test), and thus, descriptive statistics are presented as medians and IQRs. Sleep-wake patterns on scheduled and free days were compared using mixed models (lmerTest package in R).^30^

Next, we performed 3 analysis blocks, again using mixed models (lmerTest and lme4 packages in R),^30,31^ each of which was successively complemented by a further variable. To account for potential confounders, several control variables were included in all models, with sample characteristics in which the samples differed as fixed effects and school as a random effect (null model). For all models, we checked for each variable whether a random slope should be included by comparing the Akaike information criteria of the models with and without the random slope of the given variable and included the random slope if the respective Akaike information criterion was lower (variables included in each model are provided in eTable 2 in the Supplement).

First, participation in the lockdown vs the control sample was added to the null models (lockdown, 1; control, 0) to investigate differences in sleep (sleep-wake patterns, alarm clock use, and sleep problems), health-related characteristics (HRQoL and use of caffeine, alcohol, and nicotine), and digital media use. Second, to analyze associations between health and sleep, participants’ sleep period on scheduled days was added to the models with health-related characteristics as dependent variables with a significant sample main effect. Third, to investigate associations with depressive symptoms, a subanalysis was performed with the lockdown sample: the models from the analysis of associations between health and sleep were calculated with only the lockdown sample, and depressive symptoms were then added to the model.

Assumptions of regression models were visually inspected, and if needed, data were square-root transformed. Semipartial *R*^2^ statistics (*R*^2^_β*_) (determined using the r2glmm package in R)^32,33^ were reported as the effect sizes of the variables. Within analysis blocks, the significance level was Bonferroni corrected for multiple comparisons.

## Results

### Sample Characteristics

The control survey was completed by 6252 students and the lockdown survey by 4227; of these students, 944 and 563, respectively, did not meet the inclusion criteria. Thus, 8972 students (5308 [59.2%] from 17 of 20 schools in the control sample and 3664 [40.8%] from all 21 schools in the lockdown sample) were included in the analysis. Students in both samples had a median age of 16 years (IQR, 15-17 years); 3454 students [65.1%] in the lockdown sample and 2429 [66.3%] in the control sample were female (Table 1). Most (3593 [67.7%] in the control sample and 2384 [65.1%] in the lockdown sample) indicated that Swiss German was their primary language. Sex distribution and prevalence of a mental illness were comparable between the samples; however, the lockdown sample was younger compared with the control sample (mean [SD] age, 15.95 [1.87] years vs 16.09 [1.76] years) and included fewer adolescents with chronic physical diseases (196 [5.3%] vs 403 [7.6%]) and more with a primary language other than Swiss German (1280 [34.9%] vs 1681 [31.7%]. Therefore, these variables were included as control variables in the data analyses. A total of 6048 participants could be matched for characteristics in which the samples differed (ie, age, primary language, and physical disease).

**Table 1.** Characteristics of the Control and Lockdown Samples^a^

Characteristic	Participants, No. (%)		*P* value
	Control sample (n = 5308)	Lockdown sample (n = 3664)	
Age, median (IQR), y	16 (15-17)	16 (15-17)	<.001^b^
Sex			
Female	3454 (65.1)	2429 (66.3)	.24^c^
Male	1854 (34.9)	1235 (33.7)	
Swiss German native speaker	3593 (67.7)	2384 (65.1)	.003^c^
Physical disease	403 (7.6)	196 (5.3)	<.001^c^
Mental illness	276 (5.2)	185 (5.0)	.51^c^

^a^ The control sample included students who completed surveys from May to July 2017, and the lockdown sample included students who completed surveys from May to June 2020 during high school closure owing to the first COVID-19 wave in Switzerland.

^b^ Mann-Whitney *U* test was performed.

^c^ χ^2^ test was performed.

In the lockdown sample, most adolescents were in homeschooling (3412 [93.1%] yes and 64 [1.7%] no; 188 [5.1%] had data missing). A total of 2569 students (70.1%) indicated no personal or proxy COVID-19 exposure, whereas 449 (12.3%) indicated knowing at least 1 person who had been infected with COVID-19 (646 [17.6%] had data missing). Only 33 students (0.9%) indicated that they had recovered from COVID-19. Strong or very strong changes in different areas of life compared with the situation before the pandemic were reported by approximately half of the participants (strong or very strong changes in social contacts were reported by 2009 students [54.8%]; changes in digital media use by 1935 [52.8%]; and changes in sports by 1866 [50.9%]). A total of 1181 participants (32.2%) indicated that they worried strongly or very strongly about negative effects of the pandemic on their academic achievement. The median withdrawn/depressed score was 57 (IQR, 51-64), and 332 participants (9.1%) exceeded the clinical cutoff T value of 70 or greater.^29^

### Comparison of Sleep Behavior

In both samples, the adolescents’ sleep-wake patterns were significantly later and sleep period was significantly longer on free days than on scheduled days (Table 2). Large differences in sleep-wake patterns were observed between the samples (Table 3). On scheduled days, the lockdown sample slept significantly longer than the control sample (median, 9.00 hours [IQR, 8.25-9.75 hours] vs 7.75 hours [IQR, 7.08-8.33 hours]). The lockdown sample woke up 90 minutes later than the control sample (median wake time: 7:45 am [IQR, 7:10-8:27 am] vs 6:15 am [IQR, 6:00-6:30 am]) and had lower rates of alarm clock use (2735 [74.6%] vs 4587 [86.4%]). Bedtimes for the lockdown sample were 15 minutes later than for the control sample (median, 10:45 pm [IQR, 10:00-11:30 pm] vs 10:30 pm [IQR, 10:00-11:00 pm]), leading to an increase in sleep period of 75 minutes during the lockdown (*R*^2^_β*_, 0.238; 95% CI, 0.222-0.254; *P* < .001). On free days, sleep behavior of the 2 samples was comparable. The sleep deficit (ie, the difference between sleep duration on free and scheduled days) was significantly smaller in the lockdown sample.

**Table 2.** Descriptive Statistics for Self-reported Sleep-Wake Patterns on Scheduled and Free Days in the Control and Lockdown Samples^a^

Sleep characteristic	Scheduled days		Free days		Total participants, No.	*P* value	*R*^2^_β*_ (95% CI)^b^
	Participants, No.	Time, median (IQR)	Participants, No.	Time, median (IQR)			
Control sample							
Bedtime	4993	10:30 pm (10:00 to 11:00 pm)	4811	12:00 am (11:00 pm to 1:00 am)	4811	<.001	0.225 (0.211 to 0.238)
Wake time	4932	6:15 am (6:00 to 6:30 am)	4782	9:15 am (8:30 to 10:00 am)	4740	<.001	0.683 (0.674 to 0.691)
Sleep duration, h	4932	7.75 (7.08 to 8.33)	4782	9.50 (8.50 to 10.50)	4740	<.001	0.343 (0.329 to 0.357)
Lockdown sample							
Bedtime	3294	10:45 pm (10:00 to 11:30 pm)	3147	11:30 pm (10:50 pm to 12:30 am)	3140	<.001	0.085 (0.072 to 0.098)
Wake time	3275	7:45 am (7:10 to 8:27 am)	3139	9:24 am (8:30 to 10:10 am)	3122	<.001	0.267 (0.250 to 0.28)
Sleep duration, h	3275	9.00 (8.25 to 9.75)	3138	9.75 (9.00 to 10.50)	3121	<.001	0.069 (0.058 to 0.081)

^a^ The control sample included students who completed surveys from May to July 2017, and the lockdown sample included students who completed surveys from May to June 2020 during high school closure owing to the first COVID-19 wave in Switzerland.

^b^ Semipartial *R*^2^ statistic.^32^

**Table 3.** Regression Coefficients of the Sample Main Effect (Lockdown vs Control) in Models With Different Dependent Variables^a^

Dependent variable	Participants, No.	β (SE)	Corrected *P* value^b^	Uncorrected *P* value^b^	*R*^2^_β*_ (95% CI)^c^
Sleep characteristics					
Scheduled days					
Bedtime	7268	0.32 (0.04)	<.001	<.001	0.023 (0.017-0.030)
Wake time	7214	1.52 (0.09)	<.001	<.001	0.455 (0.440-0.470)
Sleep duration	7214	1.24 (0.08)	<.001	<.001	0.238 (0.222-0.254)
Free days					
Bedtime	7266	−0.14 (0.07)	>.99	.11	0.002 (0.001-0.005)
Wake time	7245	0.05 (0.05)	>.99	.31	0.000 (0.000-0.002)
Sleep duration	7244	0.17 (0.05)	.07	.005	0.004 (0.001-0.007)
Sleep deficit^d^	7193	−1.07 (0.07)	<.001	<.001	0.122 (0.108-0.136)
Alarm clock use^e^	7276	−0.69 (0.18)	.001	<.001	0.009 (0.006-0.014)
Difficulties falling asleep^f^	7276	0.16 (0.03)	<.001	<.001	0.005 (0.002-0.009)
Problems sleeping through the night^f^	7276	0.15 (0.02)	<.001	<.001	0.006 (0.003-0.011)
Health-related characteristics					
HRQoL	7243	1.47 (0.22)	<.001	<.001	0.007 (0.004-0.012)
Smoking^g^	294	−1.39 (0.63)	.41	.03	0.018 (0.001-0.059)
Alcohol consumption^h^	4562	−0.52 (0.07)	<.001	<.001	0.014 (0.008-0.022)
Caffeine consumption^i^	7276	−0.62 (0.08)	<.001	<.001	0.010 (0.006-0.015)
Digital media consumption^f^^,^^j^	7247	0.38 (0.01)	<.001	<.001	0.103 (0.091-0.117)

Abbreviation: HRQoL, health-related quality of life.

^a^ In all models, age, primary language, and physical illness were also included as fixed effects and school was included as a random effect. The control sample included students who completed surveys from May to July 2017, and the lockdown sample included students who completed surveys from May to June 2020 during high school closure owing to the first COVID-19 wave in Switzerland.

^b^ *P* values were corrected for multiple comparisons using the Bonferroni method. Uncorrected *P* values were multiplied by 15.

^c^ Semipartial *R*^2^ statistic.^32^

^d^ Calculated as free days minus scheduled days.

^e^ Logistic regression model (alarm clock use: yes vs no) using the lme4 package in R.^31^

^f^ Square-root transformed.

^g^ Only students older than 16 years who smoked were included. Smoking was measured as number of cigarettes per day.

^h^ Only students older than 16 years were included. Measured using a custom scale (eTable 1 in the Supplement) with scores ranging from 1 to 15; higher scores indicate greater alcohol consumption.

^i^ Measured using a custom scale (eTable 1 in the Supplement) with scores ranging from 1 to 20; higher scores indicate greater caffeine consumption.

^j^ Measured as hours per day.

Sleep problems were significantly more frequent in the lockdown sample than in the control sample (Table 3). Both difficulties falling asleep and problems sleeping through the night more than 4 times in the previous 2 weeks were more prevalent in the lockdown group (falling asleep: 1237 [33.8%] vs 1645 [30.9%]; problems sleeping: 437 [11.9%] vs 439 [8.3%]). However, most of the adolescents reported good sleep (ie, problems on fewer than 4 nights in the previous 2 weeks in falling asleep [1184 (51.4%)] and sleeping through the night [2869 (78.3%)]).

### Comparison of Health-Related and Behavioral Characteristics

Sample differences in health-related and behavioral characteristics were investigated (Table 3). The HRQoL scores on the KIDSCREEN-10 were significantly higher in the lockdown sample than in the control sample (median, 44.48 [IQR, 40.24-49.76] vs 42.27 [IQR, 37.42-48.29]; *R*^2^_β*_, 0.007; 95% CI, 0.004-0.012; *P* < .001). Higher values were indicated by the lockdown sample on the items for feeling fit and well, for being full of energy, for having enough time for themselves, and for being able to do the things they wanted in their free time. Conversely, adolescents in the lockdown sample indicated feeling lonelier and sadder and having less fun with friends.

Substance use was significantly lower in the lockdown sample, although only the consumption of alcohol (*R*^2^_β*_, 0.014; 95% CI, 0.008-0.022; *P* < .001) and caffeine (*R*^2^_β*_, 0.010; 95% CI, 0.006-0.015; *P* < .001) remained significant after correction for multiple comparison. Consequently, cigarette consumption was not investigated further. Digital media use in free time was significantly higher in the lockdown sample than in the control sample. Whereas adolescents in the control sample reported spending a median of 2.0 hours (IQR, 1.5-3.0 hours) per day using digital media, the lockdown sample used digital media for 3.5 hours (IQR, 2.0-5.0 hours) per day.

### Associations of Health-Related Characteristics With Sleep Period

On scheduled days, longer sleep period was associated with better HRQoL (*R*^2^_β*_, 0.027; 95% CI, 0.020-0.034; *P* < .001) and less caffeine consumption (*R*^2^_β*_, 0.013; 95% CI, 0.009-0.019; *P* < .001) (Table 4). No significant association between sleep period and alcohol consumption was found.

**Table 4.** Regression Coefficients of the Sample and Sleep Period Main Effects in Models With Different Dependent Variables^a^

Dependent variable	Participants, No.	Lockdown vs control (sample main effect)				Sleep period on scheduled days			
		β (SE)	Corrected *P* value^b^	Uncorrected *P* value^b^	R^2^_β*_ (95% CI)^c^	β (SE)	Corrected *P* value^b^	Uncorrected *P* value^b^	R^2^_β*_ (95% CI)^c^
HRQoL	7181	0.16 (0.25)	>.99	.51	0.000 (0.000-0.001)	1.28 (0.15)	<.001	<.001	0.027 (0.020-0.034)
Alcohol consumption^d^	4519	−0.48 (0.08)	<.001	<.001	0.009 (0.005-0.016)	−0.02 (0.03)	>.99	.54	0.000 (0.000-0.001)
Caffeine consumption^e^	7214	−0.26 (0.09)	.04	.01	0.001 (0.000-0.004)	−0.32 (0.04)	<.001	<.001	0.013 (0.009-0.019)

Abbreviation: HRQoL, health-related quality of life.

^a^ In all models, age, primary language, and physical illness were also included as fixed effects and school was included as a random effect. The control sample included students who completed surveys from May to July 2017, and the lockdown sample included students who completed surveys from May to June 2020 during high school closure owing to the first COVID-19 wave in Switzerland.

^b^ *P* values were corrected for multiple comparisons using the Bonferroni method. Uncorrected *P* values were multiplied by 3.

^c^ Semipartial *R*^2^ statistic.^32^

^d^ Only students older than 16 years were included. Measured using a custom scale (eTable 1 in the Supplement) with scores ranging from 1 to 15; higher scores indicate greater alcohol consumption.

^e^ Measured using a custom scale (eTable 1 in the Supplement) with scores ranging from 1 to 20; higher scores indicate greater caffeine consumption.

The between-sample difference in substance use remained similar to that in the models without sleep period, with significant differences between the samples in alcohol and caffeine consumption (Table 4). However, the between-sample difference for HRQoL was not significant when sleep period was included in the model (Table 4).

### Association of Health-Related Characteristics With Depressive Symptoms

Because depressive symptoms were assessed only in the lockdown sample, a subsample analysis was performed. First, to investigate comparability between the samples, the same models from Table 4 were calculated with only the lockdown sample. In the lockdown subsample, sleep period was positively associated with HRQoL in 3014 adolescents (β [SE], 0.48 [0.13]; *R*^2^_β*_, 0.005; 95% CI, 0.001-0.011; *P* < .001) and negatively associated with caffeine consumption in 3022 adolescents (β [SE], −0.16 [0.04]; *R*^2^_β*_, 0.004; 95% CI, 0.001-0.010; *P* < .001). There was no association between alcohol consumption and sleep period among 1787 adolescents (β [SE], 0.04 [0.04]; *R*^2^_β*_, 0.001; 95% CI, 0.000-0.005; *P* = .97).

Next, depressive symptoms were added to the models (Table 5). Significantly negative associations were found between depressive symptoms and HRQoL (*R*^2^_β*_, 0.285; 95% CI, 0.260-0.311; *P* < .001) and alcohol consumption (*R*^2^_β*_, 0.003; 95% CI, 0.000-0.011; *P* = .04), and a positive association was found with caffeine consumption (*R*^2^_β*_, 0.003; 95% CI, 0.000-0.008; *P* = .01). The associations with sleep duration remained unchanged except for HRQoL, for which no association was found when depressive symptoms were added.

**Table 5.** Regression Coefficients of the Sleep Period and YSR Withdrawn/Depressed Main Effects in Models With Different Dependent Variables^a^

Dependent variable	Participants, No.	Sleep period on scheduled days				YSR withdrawn/depressed			
		β (SE)	Corrected *P* value^b^	Uncorrected *P* value^b^	R^2^_β*_ (95% CI)^c^	β (SE)	Corrected *P* value^b^	Uncorrected *P* value^b^	R^2^_β*_ (95% CI)^c^
HRQoL	3006	0.05 (0.11)	>.99	.63	0.000 (0.000-0.002)	−0.49 (0.03)	<.001	<.001	0.285 (0.260-0.311)
Alcohol consumption^d^	1780	0.02 (0.04)	>.99	.56	0.000 (0.000-0.004)	−0.01 (0.01)	.04	.01	0.003 (0.000-0.011)
Caffeine consumption^e^	3006	−0.14 (0.05)	.01	.002	0.003 (0.000- 0.009)	0.02 (0.01)	.01	.004	0.003 (0.000-0.008)

Abbreviations: HRQoL, health-related quality of life; YSR, Youth Self Report.

^a^ In all models, age, primary language, and physical illness were also included as fixed effects and school was included as a random effect.

^b^ *P* values were corrected for multiple comparisons using the Bonferroni method. Uncorrected *P* values were multiplied by 3.

^c^ Semipartial *R*^2^ statistic.^32^

^d^ Only students older than 16 years were included. Measured using a custom scale (eTable 1 in the Supplement) with scores ranging from 1 to 15; higher scores indicate greater alcohol consumption.

^e^ Measured using a custom scale (eTable 1 in the Supplement) with scores ranging from 1 to 20; higher scores indicate greater caffeine consumption.

## Discussion

During the pandemic lockdown in spring 2020 in Switzerland, adolescents in this survey study slept a median of 75 minutes longer on school days than did adolescents under regular conditions, mainly owing to later wake times.^5,6,7,8,12,13,14^ As expected, sleep behavior on free days was comparable between samples.^5,13^ The findings suggest that school closures allowed students to better align their sleep schedules with adolescents’ late sleep phase.^16,34,35,36,37^ Of most importance, to our knowledge, this study provides the first scientific evidence for the beneficial sleep-related associations of school closures with adolescents’ health, as demonstrated by better HRQoL and decreased substance use in the lockdown sample than in the control sample. Furthermore, we found significant associations between sleep period and HRQoL and caffeine consumption. Longer sleep duration was associated with better HRQoL and less caffeine consumption. However, depressive symptoms were associated with worse HRQoL and more caffeine consumption in the lockdown sample. Thus, 2 opposing associations with adolescents’ health during school closure were identified: a beneficial sleep-related association and a detrimental distress-related association.

### Beneficial Associations With Sleep

Along with sleep gains, we observed less substance use^10^ and better HRQoL in the lockdown sample than in the control sample. This contrasts with the findings of a study from Germany,^4^ in which HRQoL was found to be lower during the lockdown than before the lockdown. The disparity might be explained by the stricter pandemic rules in Germany than in Switzerland.

The finding of an association between sleep duration and the health-related characteristics of better HRQoL and less caffeine consumption is consistent with findings of studies of SST delays.^16,19,20,21^ Of interest, when controlling for sleep period, the between-sample difference in HRQoL disappeared (Table 4), indicating that sleep duration was associated with the increase in HRQoL. In contrast, no association was found between sleep duration and alcohol consumption.

Overall, better HRQoL and less caffeine consumption appeared to be associated with increased sleep duration. Caffeine consumption and sleep are bidirectionally associated; the sleep gains owing to later SSTs may disrupt the potentially harmful cycle of consuming more caffeine owing to insufficient sleep, which in turn is associated with even worse sleep.^16,20^ The adolescents in lockdown not only slept longer and in better alignment with their late sleep phase but also showed more regular sleep-wake patterns across the week, which is known to be beneficial for adolescents’ health.^38^ A possible mechanism for the association between sleep and HRQoL may be the direct association of sleep with mental, social, and physical functioning.^39^ Numerous studies^40^ have shown that sleep deficits and insomnia are associated with substantially impaired coping in everyday life; declines in mood and cognitive functioning such as concentration, memory, and attention; and general fatigue, anxiety, and physical discomfort.

### Detrimental Associations With Psychological Distress

This study adds to evidence of psychological distress among adolescents during the COVID-19 lockdown^1,2,3,4^; clinically relevant levels of depressive symptoms were found in 9.1% of adolescents in lockdown, 4.5 times more prevalent than the expected level of 2%.^2,29^ Although total HRQoL was better during lockdown, comparison of individual items on the KIDSCREEN-10 showed that adolescents in lockdown felt lonelier and sadder and had less fun with friends. Adolescents indicated high perceived changes in different areas of life, and 32.2% of students indicated that they worried strongly or very strongly about negative consequences of the pandemic on their future academic achievement—a finding that should be followed up in future studies. In addition, digital media use was higher in the lockdown sample,^5,6,7,8,9^ and, potentially associated with this,^16^ sleep problems were more frequent during lockdown.^5,13^ Thus, the longer sleep periods may have been a consequence of worse sleep quality, such as fragmented sleep, which necessitated longer sleep duration to compensate. Assessment of sleep quality was not possible with our approach. However, the between-group difference in sleep duration was more pronounced than the difference in sleep problems, and most adolescents reported good sleep.

The positive associations of health-related characteristics with sleep coexisted with negative associations with depressive symptoms. Significant associations of depressive symptoms with worse HRQoL and more caffeine consumption were apparent, likely related to the overall association between depressive symptoms and health-compromising behaviors.^41^ Whereas caffeine consumption and sleep duration were still significantly associated when depressive symptoms were added to the model, there was no association between HRQoL and sleep duration, suggesting the detrimental association between depressive symptoms and HRQoL was stronger than the beneficial association between increased sleep duration and HRQoL. However, the data did not allow a precise weighting of the 2 associations identified, and further research is needed to investigate the interplay between them; those findings may be helpful for future policy decisions.

### Limitations

This study has limitations. The most important limitation is its pseudolongitudinal approach: the 2 samples consisted of students from mostly the same schools, and it is likely that some students participated in both samples. However, because the surveys were answered anonymously, quantification or even paired analysis was not possible. Therefore, the data only allowed investigation of associations between measures, not the investigation of associations among changes or causality. Also, the samples differed in some characteristics (ie, age, primary language, and physical disease) that are difficult to interpret. However, a subgroup analysis with 6048 participants matched for those characteristics confirmed the results presented in this article. Furthermore, 3 years passed between the surveys; thus, potential cohort effects cannot be excluded. For instance, the greater use of digital media in the lockdown sample may be explained by progressive digitalization, and a regressive trend was observed in alcohol use.^42^ No details about homeschooling were available for the lockdown sample, such as whether virtual lessons took place at fixed times, whether only flexible self-study occurred, or whether students participated in in-person lessons of a maximum of 5 people starting from May 11, 2020. Moreover, generalizability is limited because Switzerland applied only partial lockdown measures^24^ and because survey participation rates were low, with a selection bias of female students; however, these rates are likely to be underestimates because the number of students who eventually received the study invitation is unknown.

## Conclusion

In this survey study, adolescents in the lockdown sample slept more than 1 hour longer on school days than did adolescents in the control sample, and sleep duration was associated with better health-related characteristics. Concurrently, depressive symptoms were associated with poorer HRQoL and increased caffeine consumption and thus diminished the beneficial associations with sleep. Thus, evaluation of school closures should be supplemented by these observed positive associations, and the beneficial sleep associations that may counteract the negative outcomes should be considered when implementing school closures. Furthermore, this study provides further support for delaying SSTs in countries with early SSTs.

## Notes

Supplement. **eTable 1.** All Questions and Answer Formats or Response Scales **eTable 2.** Predictors Included in the Mixed Models
