# Supplementary material for: Association Between Homeschooling and Adolescent Sleep Duration and Health During COVID-19 Pandemic High School Closures
Source: JAMA Netw Open. 2022 Jan 5;5(1):e2142100. doi: 10.1001/jamanetworkopen.2021.42100 (PMC8733832; doi:10.1001/jamanetworkopen.2021.42100)
Supplement: Supplement. — eTable 1. All Questions and Answer Formats or Response Scales eTable 2. Predictors Included in the Mixed Models [file jamanetwopen-e2142100-s001.pdf]

Supplementary Online Content

Albrecht JN, Werner H, Rieger N, et al. Association between homeschooling and adolescent sleep duration and health during COVID-19 pandemic high school closures. *JAMA Netw Open*. 2022;5(1):e2142100. doi:10.1001/jamanetworkopen.2021.42100

eTable 1. All Questions and Answer Formats or Response Scales
eTable 2. Predictors Included in the Mixed Models

This supplementary material has been provided by the authors to give readers additional information about their work.

© 2022 Albrecht JN et al. *JAMA Network Open*.

Supplementary Material

Questionnaire

All questions listed below in eTable 1 were originally in German. If not specified differently, the questions were the same for the control and the lockdown samples.

eTable 1. All questions and answer formats or response scales.

	Variable	*Question*	*Answer format or response scale*
**Sample characteristics**	Biological sex	Your sex?	Male / female
	Age	Your age?	In years
	Primary language	What is your primary language?	Swiss German / German / else
	Physical disease	Do you suffer from a physical disease?	Yes / no
	Mental disease	Are you currently in therapy because of a psychiatric disorder?	Yes / no
	School	To which school do you go?	Selection of one of the 21 schools
**Sleep characteristics**	Bedtime on SC days	Control sample: When do you normally go to bed on scheduled days (school days)?<br>Lockdown sample: When do you normally go to bed on scheduled days (homeschooling days)?	Hours:minutes
	Wake time on SC days	Control sample: When do you normally wake up on scheduled days (school days)?<br>Lockdown sample: When do you normally wake up on scheduled days (homeschooling days)?	Hours:minutes
	Bedtime on SC days	When do you normally go to bed on free days (weekends, holidays)?	Hours:minutes
	Wake time on SC days	When do you normally wake up on free days (weekends, holidays)?	Hours:minutes
	Alarm clock usage	I use an alarm clock on scheduled days	Yes / no
	Difficulties falling asleep	How often did you have difficulties falling asleep in the past 14 days?	Never – 14x
	Difficulties sleeping through	How often did you have difficulties sleeping through the night in the past 14 days?	Never – 14x

© 2022 Albrecht JN et al. *JAMA Network Open*.

	Variable	*Question*	*Answer format or response scale*
**Health-related and behavioural characteristics**	Health-related quality of life	KIDSCREEN-10[1]	
	Depressive symptoms	Only in lockdown sample: Youth Self Report withdrawn/depression scale[2]	
	Caffeine consumption	On how many days (incl. the weekend) do you consume the following drinks?[3]	
		Coffee (e.g. cappuccino, espresso)	Every day (1) / almost every day / several times per week / once a week / rarer / never (5)
		Energy drinks (e.g. Red Bull)	Every day (1) / almost every day / several times per week / once a week / rarer / never (5)
		Coca-Cola-like sweet drinks (e.g. Coca-Cola, Pepsi, Afri-Cola)	Every day (1) / almost every day / several times per week / once a week / rarer / never (5)
		Black, white, or green tea	Every day (1) / almost every day / several times per week / once a week / rarer / never (5)
	Alcohol consumption	On how many days (incl. the weekend) do you consume the following drinks?[4]	
		Beer	Every day (1) / almost every day / several times per week / once a week / rarer / never (5)
		Wine	Every day (1) / almost every day / several times per week / once a week / rarer / never (5)
		Spirits (such as gin, whiskey, …)	Every day (1) / almost every day / several times per week / once a week / rarer / never (5)
	Smoking	Do you smoke?	Yes / no
	Number of cigarettes[5]	How many cigarettes do you smoke on a typical weekday?	Number of cigarettes
		How many cigarettes do you smoke on a typical weekend?	Number of cigarettes
	Digital media use	Control sample: How much time do you usually spend on scheduled days with digital media (smartphone, iPad, tablet, PC, etc.)?<br>Lockdown sample: How much time do you usually spend on scheduled days with digital media (smartphone, iPad,	Hours:minutes

© 2022 Albrecht JN et al. *JAMA Network Open*.

	Variable	*Question*	*Answer format or response scale*
		tablet, PC, etc.)? Please only refer to the time spent with digital media in your free time.	
**COVID-19-related measures (lockdown sample only)**	Homeschooling	Do the lessons of your class currently take place in homeschooling?	Yes / no
	Changes in social contacts	How much did my social contacts with friends or other people change (e.g. frequency, manner, duration)?	1: not at all – 5: very strongly
	Changes in use of digital media	How much did my digital media use in my free time change (e.g. Netflix, Youtube, gaming with smartphone, iPad, tablet, PC) compared to before the corona pandemic?	1: not at all – 5: very strongly
	Changes in sports	How much did my sports activities change compared to before the corona pandemic?	1: not at all – 5: very strongly
	Worry about future academic achievement	I worry that the pandemic will negatively impact my future academic achievement.	1: not at all – 5: very strongly
	COVID-19 exposure - self	I suffer from corona?	No / Yes, currently / Yes, recovered
	COVID-19 exposure - household	Does someone in your household suffer from corona?	No / Yes, currently / Yes, recovered
	COVID-19 exposure - someone close	Does someone you're close with (e.g. grandparent, friend) suffer from corona?	No / Yes, currently / Yes, recovered

Notes:

[1] Ravens-Sieberer U, Erhart M, Rajmil L, et al. Reliability, construct and criterion validity of the KIDSCREEN-10 score: A short measure for children and adolescents' well-being and health-related quality of life. *Qual Life Res*. 2010;19(10):1487-1500. doi:10.1007/s11136-010-9706-5

[2] Döpfner M, Plück J, Kinnen C. *Für Die Arbeitsgruppe Deutsche Child Behavior Checklist. Manual Deutsche Schulalter-Formen Der Child Behavior Checklist von Thomas M. Achenbach. Elternfragebogen Über Das Verhalten von Kindern Und Jugendlichen*. Göttingen: Hogrefe; 2014.

[3] The answer scores for all four beverages were summed up, leading to a total score of 0–20 on which higher scores indicate greater weekly caffeine consumption.

[4] The answer scores for all three beverages were summed up, leading to a total score of 0–15 on which higher scores indicate greater weekly alcohol consumption.

[5] The answers of the two questions were afterwards combined into one single measure of weekly cigarette consumption.

© 2022 Albrecht JN et al. *JAMA Network Open*.

eTable 2. Predictors included in the mixed models.

	Dependent variable	*Fixed effects*	*Random effect*	*Random slopes*
**Sample differences in sleep**	Bedtime on SC days	Sample, age, primary language, physical disease	School	Sample, primary language
	Bedtime on FR days	Sample, age, primary language, physical disease	School	Sample, primary language, physical disease
	Wake time on SC days	Sample, age, primary language, physical disease	School	Sample, age, primary language
	Wake time on FR days	Sample, age, primary language, physical disease	School	Sample
	Sleep period on SC days	Sample, age, primary language, physical disease	School	Sample, age
	Sleep period on FR days	Sample, age, primary language, physical disease	School	Sample
	Sleep deficit	Sample, age, primary language, physical disease	School	Sample, physical disease
	Difficulties falling asleep (square-root transformed)	Sample, age, primary language, physical disease	School	Age
	Difficulties sleeping through (square-root transformed)	Sample, age, primary language, physical disease	School	Age
	Alarm clock usage on SC days	Sample, age, primary language, physical disease	School	Sample
**Sample differences in healthy-related and behavioural characteristics**	Health-related quality of life	Sample, age, primary language, physical disease	School	-
	Alcohol consumption	Sample, age, primary language, physical disease	School	Age
	Smoking (# cigarettes per day)	Sample, age, primary language, physical disease	School	-
	Caffeine consumption	Sample, age, primary language, physical disease	School	Primary language
	Digital media use (square-root transformed)	Sample, age, primary language, physical disease	School	-

© 2022 Albrecht JN et al. *JAMA Network Open*.

	Dependent variable	*Fixed effects*	*Random effect*	*Random slopes*
**Associations of sleep period and health-related characteristics**	Health-related quality of life	Sample, sleep period, age, primary language, physical disease	School	Sleep period
	Alcohol consumption	Sample, sleep period, age, primary language, physical disease	School	Age
	Caffeine consumption	Sample, sleep period, age, primary language, physical disease	School	Sample, sleep period
**Only lockdown sample: Associations of sleep period and health-related characteristics**	Health-related quality of life	Sleep period, age, primary language, physical disease	School	-
	Alcohol consumption	Sleep period, age, primary language, physical disease	School	Age
	Caffeine consumption	Sleep period, age, primary language, physical disease	School	-
**Only lockdown sample: Associations of YSR withdrawn/depression and health-related characteristics**	Health-related quality of life	Sleep period, YSR withdrawn/depression, age, primary language, physical disease	School	YSR withdrawn/depression
	Alcohol consumption	Sleep period, YSR withdrawn/depression, age, primary language, physical disease	School	Age
	Caffeine consumption	Sleep period, YSR withdrawn/depression, age, primary language, physical disease	School	-

© 2022 Albrecht JN et al. *JAMA Network Open*.
